# Supplementary material for: Basal ganglia-cortical connectivity underlies self-regulation of brain oscillations in humans
Source: Commun Biol. 2022 Jul 16;5:712. doi: 10.1038/s42003-022-03665-6 (PMC9288463; doi:10.1038/s42003-022-03665-6)
Supplement: Supplementary file 2 — Supplementary Information [file 42003_2022_3665_MOESM2_ESM.pdf]

Supplementary Information

Basal ganglia-cortical connectivity underlies successful self-regulation of brain oscillations in humans

Kazumi Kasahara, Charles S. DaSalla, Manabu Honda, and Takashi Hanakawa*

*Corresponding author. Email: hanakawa.takashi.2s@kyoto-u.ac.jp

This PDF file includes:

Supplementary Note 1
Supplementary Figure 1
Supplementary Figure 2
Supplementary Table 1
Supplementary Table2

Supplementary Note 1

Nonsignificant effects on BCI performance
Motor imagery ability, which was assessed using the revised Movement Imagery Questionnaire, showed no correlation with hit rate ($r = 0.01$, $P = 0.96$). Neither sex ($\chi^2 = 22$, $P = 0.40$) nor age ($r = 0.14$, $P = 0.52$) significantly affected hit rate.

SMR changes and muscle activity during BCI control
To compare hit rate with changes in sensorimotor rhythms (SMRs), inMRI EEG data were analyzed offline, and the spectral amplitude (2–23-Hz, 3-Hz bins) was extracted from C3 and C4 electrodes during the 4-s BCI control period. During right target (RT) trials, most participants showed peak event-related desynchronization (ERD) at C3 within either the 5-Hz bin or 11-Hz bin. During left target (LT) trials, most participants showed peak ERD at C4 within the 11-Hz bin. Percent difference from amplitude during the rest was also highest within the 11-Hz bin (**Fig. S1a**). Spectral data averaged across subjects (**Fig. S1b**) showed that decreases in SMR amplitude, namely ERD, mostly occurred within the 11-Hz bin (9.5–12.5 Hz). Moreover, hit rates were correlated with the laterality of SMR ERD during the inMRI session (**Fig. S1c**). These results suggest that the control strategies during the inMRI session were essentially the same as those employed during the outMRI session, despite the noisier and less-comfortable MRI environment.

To rule out the potential influence of overt hand/ocular movements on BCI performance, time series data for vertical and horizontal electrooculogram (EOG) and electromyography (EMG) from the bilateral thenar muscles were analyzed. The root mean squares of EOG and EMG were calculated from the 4-s BCI control periods and compared between the tasks. No statistical differences were found between LT, RT, and rest tasks for any EOG or EMG channels (repeated measures ANOVA, $P > 0.1$ for each channel). These results excluded the possibility that differences in BCI performance/brain activity resulted from muscle activation or ocular movements.

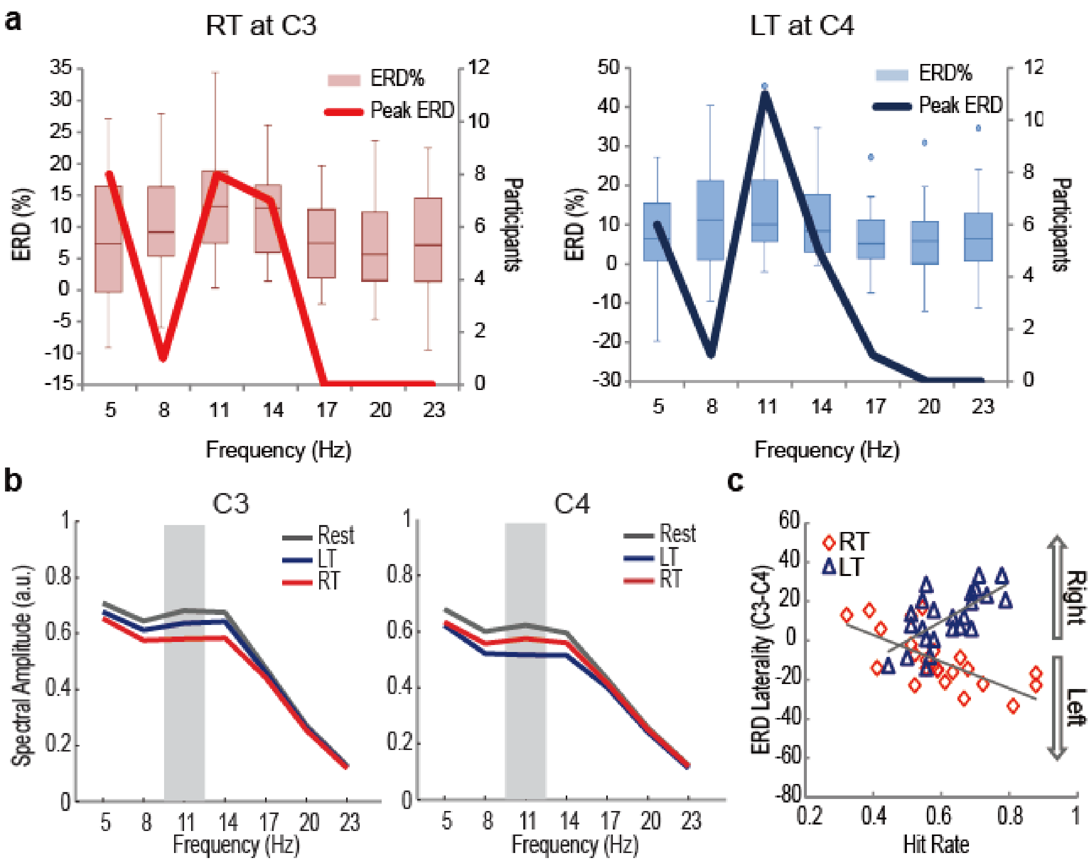

Supplementary Figure 1 Event-related desynchronization (ERD) during BCI control inside the MRI scanner. (**a**) ERD percentages (boxplots) and histogram of peak ERD frequencies (lines) at C3 for the right target (RT) (red) and C4 for the left target (LT) (blue). The center lines of the boxplots indicate the medians, box limits indicate the lower and upper quartiles, the whiskers represent 1.5 times interquartile range, and circle indicates the outlier. (**b**) EEG spectral amplitudes at C3 and C4 averaged across participants. Gray shaded areas indicate 11-Hz bins (9.5–12.5-Hz band). (**c**) Correlation between hit rate and ERD laterality (C3-ERD percentage minus C4-ERD percentage) for RT and LT trials. a.u., arbitrary units.

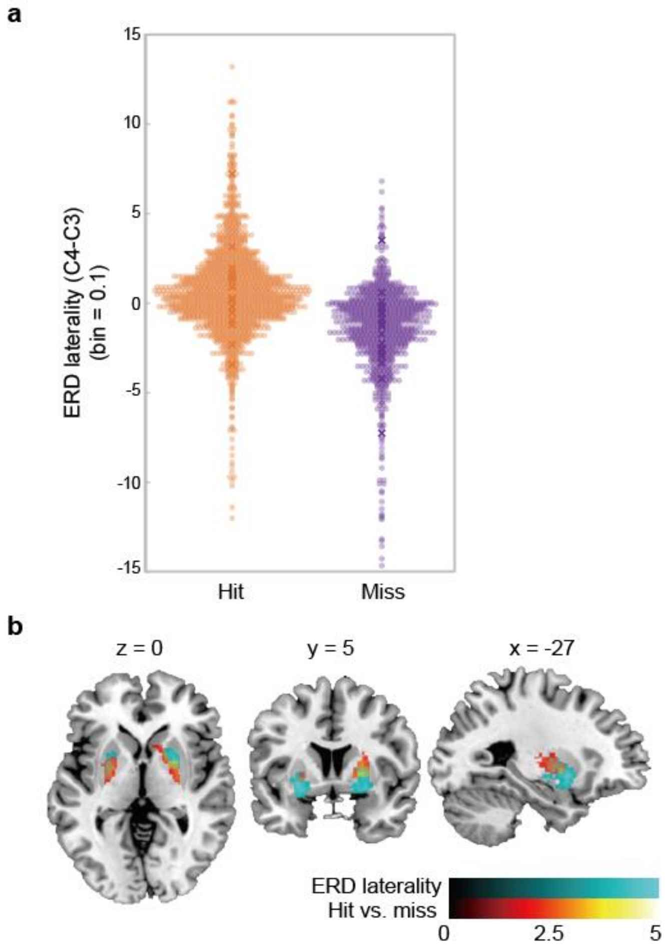

Supplementary Figure 2. Trial-by-trial ERD laterality. A. Trial-by-trial variation of the ERD laterality in all trials for all participants. Each circle indicates the ERD laterality in each trial in each participant. The crosses indicate the mean ERD laterality in each participant. The ERD laterality was computed from raw EEG data before any weighting or calibration by BCI2000. **B**. Correlation between the trial-by-trial ERD laterality and brain activity during the BCI control period for the right target (light blue). This activity overlapped with the putamen that were more activated in the hit than the miss trials shown in red. The yellow areas indicate the overlap of the two activities.

Supplementary Table 1
Hit rates for inMRI and outMRI sessions

Subject		inMRI		outMRI	
		Hit rate	*P* value	Hit rate	*P* value
S01	F (22)	0.80	8.42E-17**	0.91	1.94E-21**
S02	F (24)	0.79	3.31E-16**	0.86	3.41E-16**
S03	F (22)	0.71	6.98E-09**	0.80	2.16E-11**
S04	M (21)	0.71	1.72E-08**	0.74	1.12E-07**
S05	F (21)	0.69	9.59E-08**	0.92	1.92E-22**
S06	M (22)	0.66	9.21E-06**	0.93	1.71E-23**
S07	M (34)	0.64	6.52E-05**	0.69	3.24E-05**
S08	M (20)	0.64	0.00012**	0.78	3.14E-10**
S09	M (20)	0.63	0.00038**	0.80	2.16E-11**
S10	F (21)	0.62	0.00064**	0.69	3.24E-05**
S11	F (22)	0.61	0.00177**	0.85	1.98E-15**
S12	M (23)	0.61	0.00283**	0.83	2.66E-13**
S13	F (20)	0.59	0.00684**	0.61	0.02208*
S14	F (22)	0.58	0.01518*	0.70	1.39E-05**
S15	F (20)	0.55	0.10250 NS	0.76	1.22E-08**
S16	M (26)	0.54	0.13175 NS	0.63	0.00785**
S17	F (23)	0.54	0.16629 NS	0.63	0.00446**
S18	F (20)	0.53	0.20618 NS	0.67	0.00033**
S19	M (25)	0.53	0.25122 NS	0.48	1.35173 NS
S20	F (21)	0.53	0.25122 NS	0.53	0.64826 NS
S21	M (23)	0.51	0.47031 NS	0.60	0.03532*
S22	M (21)	0.50	0.52969 NS	0.66	0.00067**
S23	M (20)	0.50	0.52969 NS	0.63	0.00446**
S24	M (20)	0.45	0.92173 NS	0.47	1.58857 NS

**, $P < 0.005$; *, $P < 0.05$; NS, not significant by two-tailed exact binomial test

Supplementary Table 2

Areas that exhibited task-related activation and their coordinates

Area	Cluster	x	y	z	*t* value
**LT**					
Cluster-level *P* < 0.05 FWE corrected					
Bilateral anterior insula cortex, basal ganglia, thalamus, cerebellum	16,251	-32	22	2	9.89
Bilateral supplementary motor area, premotor cortex	6,279	-2	-4	66	8.98
Right inferior parietal lobule	781	52	-34	34	5.76
Left inferior parietal lobule	580	-36	-42	42	5.56
Uncorrected *P* < 0.001, >30 voxels; bilateral dorsolateral prefrontal cortex, bilateral superior parietal lobule, right lateral occipital cortex					
RT					
Cluster-level *P* < 0.05 FWE corrected					
Left basal ganglia	17,548	-24	2	2	10.54
Left supplementary motor area, premotor cortex	3854	-12	-8	74	9.8
Right- superior parietal lobule, precuneus	607	12	-62	64	5.92
Right inferior parietal lobule	587	-54	-36	26	5.41
Right lateral occipital cortex	404	66	-30	38	4.68
Uncorrected *P* < 0.001, > 30 voxels; bilateral dorsolateral prefrontal cortex					
LT and RT					
Cluster-level *P* < 0.05 FWE corrected					
Bilateral anterior insula cortex, basal ganglia, thalamus, cerebellum, supplementary motor area, premotor cortex	14,989	32	18	6	12.19
Left cerebellum	1336	-20	-62	-24	8.65
Right inferior parietal lobule	1132	62	-26	40	8.16
Left inferior parietal lobule	930	-62	-34	30	6.98
Right cerebellum	532	32	-50	-34	6.77
Left precuneus, superior parietal lobule	254	-14	-56	65	6.18
Uncorrected *P* < 0.001, >30 voxels; bilateral lateral occipital cortex, right superior parietal lobule					
LT > RT					
Cluster-level *P* < 0.05 FWE corrected					
Right visual cortex	698	20	-94	20	8.48
Right primary motor cortex	1,304	26	-22	76	5.52
Left cerebellum	466	-18	-50	-22	5.28
LT < RT					
Cluster-level *P* < 0.05 FWE corrected					
Left visual cortex	1,133	-14	-96	18	7.39
Right lingual, cerebellum	1,712	8	-68	-2	6.20
Left primary motor cortex	784	-40	-30	50	4.47
Uncorrected *P* < 0.001, >30 voxels; left putamen					
