# Supplementary material for: Basal ganglia-cortical connectivity underlies self-regulation of brain oscillations in humans
Source: Commun Biol. 2022 Jul 16;5:712. doi: 10.1038/s42003-022-03665-6 (PMC9288463; doi:10.1038/s42003-022-03665-6)
Supplement: Supplementary file 3 — Description of Additional Supplementary Files [file 42003_2022_3665_MOESM3_ESM.pdf]

Description of Additional Supplementary Files

File name: Supplementary Data 1
Description: The source data behind the graphs in Fig. 2

File name: Supplementary Data 2
Description: The source data behind the graphs in Fig. 3a

File name: Supplementary Data 3
Description: The source data behind the graphs in Fig. 3b

File name: Supplementary Data 4
Description: The source data behind the graphs in Fig. 5b
