# Supplementary material for: Basal ganglia-cortical connectivity underlies self-regulation of brain oscillations in humans
Source: Commun Biol. 2022 Jul 16;5:712. doi: 10.1038/s42003-022-03665-6 (PMC9288463; doi:10.1038/s42003-022-03665-6)
Supplement: Supplementary file 8 — Reporting Summary [file 42003_2022_3665_MOESM8_ESM.pdf]

nature research

Corresponding author(s): Takashi Hanakawa

Last updated by author(s): Jun 7, 2022

Reporting Summary

Nature Research wishes to improve the reproducibility of the work that we publish. This form provides structure for consistency and transparency in reporting. For further information on Nature Research policies, see our Editorial Policies and the Editorial Policy Checklist.

Statistics

For all statistical analyses, confirm that the following items are present in the figure legend, table legend, main text, or Methods section.

n/a	Confirmed	
☐	☒	The exact sample size (*n*) for each experimental group/condition, given as a discrete number and unit of measurement
☐	☒	A statement on whether measurements were taken from distinct samples or whether the same sample was measured repeatedly
☐	☒	The statistical test(s) used AND whether they are one- or two-sided<br>*Only common tests should be described solely by name; describe more complex techniques in the Methods section.*
☐	☒	A description of all covariates tested
☐	☒	A description of any assumptions or corrections, such as tests of normality and adjustment for multiple comparisons
☐	☒	A full description of the statistical parameters including central tendency (e.g. means) or other basic estimates (e.g. regression coefficient) AND variation (e.g. standard deviation) or associated estimates of uncertainty (e.g. confidence intervals)
☐	☒	For null hypothesis testing, the test statistic (e.g. *F*, *t*, *r*) with confidence intervals, effect sizes, degrees of freedom and *P* value noted<br>*Give P values as exact values whenever suitable.*
☒	☐	For Bayesian analysis, information on the choice of priors and Markov chain Monte Carlo settings
☒	☐	For hierarchical and complex designs, identification of the appropriate level for tests and full reporting of outcomes
☐	☒	Estimates of effect sizes (e.g. Cohen's *d*, Pearson's *r*), indicating how they were calculated

Our web collection on statistics for biologists contains articles on many of the points above.

Software and code

Policy information about availability of computer code

Data collection	EEG: Vision Recorder (www.BrainProducts.com)
Data analysis	EEG:BCI2000 (National Center for Adaptive Neurotechnologies, http://www.schalklab.org/research/bci2000), MATLAB 2015b (MathWorks https://www.mathworks.com)<br>MRI: SPM (Wellcome Department of Cognitive Neurology, London, UK, http://www.fil.ion.ucl.ac.uk/spm/), FSL (FMRIB Software Library, https://fsl.fmrib.ox.ac.uk/fsl), MATLAB 2015b (MathWorks https://www.mathworks.com), Python (Python Software Foundation, https://www.python.org)

For manuscripts utilizing custom algorithms or software that are central to the research but not yet described in published literature, software must be made available to editors and reviewers. We strongly encourage code deposition in a community repository (e.g. GitHub). See the Nature Research guidelines for submitting code & software for further information.

Data

Policy information about availability of data

All manuscripts must include a data availability statement. This statement should provide the following information, where applicable:

- Accession codes, unique identifiers, or web links for publicly available datasets
- A list of figures that have associated raw data
- A description of any restrictions on data availability

The datasets generated during and/or analyzed during the current study are available from the corresponding author on request

Field-specific reporting

Please select the one below that is the best fit for your research. If you are not sure, read the appropriate sections before making your selection.

☒ Life sciences ☐ Behavioural & social sciences ☐ Ecological, evolutionary & environmental sciences

For a reference copy of the document with all sections, see nature.com/documents/nr-reporting-summary-flat.pdf

Life sciences study design

All studies must disclose on these points even when the disclosure is negative.

Sample size	n = 26 for the simultaneous EEG-BCI (Brain-Computer Interface) and fMRI measurements.<br>n= 15 only for defining the VOI (We re-used the another subjects' DTI data published in another paper (Hanakawa, eNeuro, 2017).)
Data exclusions	Excluded our data from two participants with excessive EEG artifacts
Replication	Multiple analyses (i.e. Figure 3A and 3B, 4A and 4B, 5A and 5B) assessed robustness and consistency of our main findings.
Randomization	N/A<br>The participants no allocated the groups.
Blinding	N/A

Reporting for specific materials, systems and methods

We require information from authors about some types of materials, experimental systems and methods used in many studies. Here, indicate whether each material, system or method listed is relevant to your study. If you are not sure if a list item applies to your research, read the appropriate section before selecting a response.

Materials & experimental systems

n/a	Involved in the study	
☒	☐	Antibodies
☒	☐	Eukaryotic cell lines
☒	☐	Palaeontology and archaeology
☒	☐	Animals and other organisms
☐	☒	Human research participants
☒	☐	Clinical data
☒	☐	Dual use research of concern

Methods

n/a	Involved in the study	
☒	☐	ChIP-seq
☒	☐	Flow cytometry
☐	☒	MRI-based neuroimaging

Human research participants

Policy information about studies involving human research participants

Population characteristics	Subjects without neurological or psychiatric disease (22.4 ± 2.9 years)
Recruitment	Recruitment was randomly invited using an advertisement describing the details of the experiments posted on the university bulletin board.
Ethics oversight	National Center of Neurology and Psychiatry

Note that full information on the approval of the study protocol must also be provided in the manuscript.

Magnetic resonance imaging

Experimental design

Design type	Task (EEG-based BCI control), block design, simultaneous EEG and fMRI
Design specifications	Number of session: 3 session<br>Number of block per session: 31 blocks (10 Left, 10 Right, and 11 Rest)<br>Trial per block: 3 trials<br>Trial per session: 3trials x 31blocks = 93 (30 Left, 30 Right, and 33 Rest)<br>Length of each trial: 6 sec (1s for visual stimuli, 4s for BCI control, and 1s for outcome)

Interval between trials: 1 sec

For the task that controlled BCI used in this study, visual stimuli, feature extraction, and classification were all performed using the BCI2000 software platform. Participants were asked to perform two motor imagery tasks: imagery of finger-thumb opposition with the left and right hands, and a baseline "rest" task. For the imagery tasks, participants were instructed to use, to the best of their ability, a first-person perspective and kinesthetic rather than visual imagery.

Behavioral performance measures

To evaluate BCI performance, the hit rate was calculated as the number of times the cursor hit the left or right target divided by the number of imagery trials in each run for each participant. The hit rate was calculated over all three runs, and the overall significance was compared with chance (58%, $P < 0.05$, two-tailed exact binomial test). BCI performance was pooled from three inMRI runs, as no differences in hit rate were found between runs ($F(1.9, 43.5) = 1.86$, $P = 0.17$).

Acquisition

Imaging type(s)

Functional

Field strength

3-T

Sequence & imaging parameters

Sequence: T2*-weighted gradient echo, echo planar imaging
TR = 3 s
TE = 30 ms
Flip angle = 90°
Voxel size = 3.0 mm3 isotropic
Number of slices = 42 slices
Total scans = 262 scans

Area of acquisition

whole brain

Diffusion MRI ☒ Used ☐ Not used

Parameters

We used Diffusion MRI for defining VOI using our previous study (Hanakawa, eNeuro, 2017).

Diffusion Weighted Imaging (DWI) with twice-refocused, single-shot, spin-echo echo planar imaging (TR = 7900 ms, TE = 80 ms, FA = 90°, slice thickness = 2 mm, matrix size = 96 × 68, FOV = 192 × 192 mm, 68 axial slices).
A single acquisition included 81 DWIs (b-value = 1000 s/mm2 with different motion-probing gradient directions) and nine non-DWIs (b-value = 0 s/mm2).

Preprocessing

Preprocessing software

SPM8 (http://www.fil.ion.ucl.ac.uk/spm)

Normalization

All fMRI data preprocessing and analyses were performed using SPM8. The functional images underwent slice-timing correction and spatial realignment. The realigned images were then normalized to the Montreal Neurological Institute stereotactic space using the standard echo planar imaging template in SPM8. Finally, the normalized images were spatially smoothed using a Gaussian kernel of 6-mm full-width at half-maximum.

Normalization template

The Montreal Neurological Institute space

Noise and artifact removal

For all designs, data were high-pass filtered (1/128-Hz cutoff) to remove low-frequency drift, and realignment parameters acquired during preprocessing were included to regress out head movement artifacts.

Volume censoring

N/A

Statistical modeling & inference

Model type and settings

BCI performance: Pearson correlation
Functional images: one sample t-tests (random effect model)
Psychophysiological interaction analysis: one sample t-tests (random effect model), Lasso regression (using python)

Effect(s) tested

Fisher's Exact Test in fMRI time course in Figure3B

Specify type of analysis: ☐ Whole brain ☐ ROI-based ☒ Both

Anatomical location(s)

ROI in Figure 3B: Previously published atlases (Hanakawa, eNeuro, 2017)

ROI in Figure 5B: 10-mm spheric VOI set at the peak coordinate of each cluster in the task-fMRI analysis

Statistic type for inference
(See Eklund et al. 2016)

Voxel wise

Correction

Whole brain: FWE correction at the cluster level
ROI-based: t-test

Models & analysis

n/a	Involved in the study
☐	☒ Functional and/or effective connectivity
☒	☐ Graph analysis
☒	☐ Multivariate modeling or predictive analysis

Functional and/or effective connectivity

The ventral striatum that was correlated with BCI performance was set as seed, and effective connectivity (psychophysiological interaction) during the task (BCI control) was calculated.
